# Isolation of *Hymenolepis diminuta* cysticercoids for therapeutic use: The need for fully disclosed methodology

**DOI:** 10.12688/f1000research.186809.2

**Published:** 2026-09-17

**Authors:** Aarushi Venkatakrishnan, Chelsea Swanson, Antara Palkar, David Takamiya, Joshua T. Sarafian, Jennifer Mauney, Margaret W. Gatongi, Jasmine Cendejas Hernandez, Victoria Lawton, Zoie E. Holzknecht, John P. Jones III, Zacharoula Konsoula, Kateřina Jirků, Garin Aglietti, Detlev Goj, William Parker

**Affiliations:** 1 Surgery, Duke University Medical Center, Durham, NC, 27710, USA; 2 WPLab, Durham, NC, 27705, USA; 3 Duke Pratt School of Engineering, Durham, NC, USA; 4 Institute of Parasitology, Czech Academy of Sciences, Branišovská, Czech Republic; 5 Tanawisa Company Ltd, Pranakornsriayuthaya, Thailand; 6 Psychology and Neuroscience, The University of North Carolina at Chapel Hill, Chapel Hill, North Carolina, USA

**Keywords:** allergic, autoimmune, cestode, helminth therapy, purification

## Abstract

**Background:**

Helminth therapy holds considerable theoretical promise for clinical and public health applications. Unfortunately, clinical trials have often used helminths produced using methods that are both highly variable and insufficiently described in the literature, which may hinder reproducibility and troubleshooting. This study aimed to devise a method for purification of helminths that avoids mechanical filtration, which may damage some organisms. In particular, the goal was to devise a method to separate large quantities of cysticercoids of the cestode species *Hymenolepis diminuta* (HDCs), a process that has not been described previously but that would be necessary before HDCs can be used clinically.

**Methods:**

The physical size and behavior of HDCs in solution were assessed and used to create a patented washing device.

**Results:**

The cross-sectional area of HDCs varied over a 10-fold range, from approximately 0.06 to 0.6 mm ^2^, dependent in large part upon the host. That cross-sectional area correlated in a linear fashion with the steady-state speed of HDCs while falling through solution, which varied over a 5-fold range, from approximately 0.4 mm/s to 2.0 mm/s in the solution tested. These parameters were used to develop a washing device that utilized a variable flow speed dictated by changing column diameter and an upward flowing column of liquid that effectively and simultaneously suspended HDCs of various sizes in a wash solution.

**Conclusions:**

This study describes a detailed method of purification of HDCs, organisms that could potentially be used for helminth therapy. It is hoped that government agencies or non-profit organizations can use such technology to conduct rigorous clinical trials, potentially leading to the production of reasonably priced therapeutics.

## Introduction

The concept of “biota restoration” involves the reintroduction of organisms into the ecosystem of the human body which have been lost as a result of “systems hygiene”, or changes imposed by human civilization aimed at preventing infectious disease. ^1^ These changes have resulted in the loss of contact with a variety of symbiotic organisms, primarily various types of helminths, including roundworms (nematodes) and tapeworms (cestodes). Health problems associated with this loss, termed biota depletion, include allergic, autoimmune and neuropsychiatric conditions. ^2, 3^ Helminth therapy, therefore, serves as a first step in biota restoration by treating individuals with immunological problems associated with biota depletion. In addition, some helminths may attenuate the development of colon cancer under some conditions. ^4– 7^ However, progress in the field of helminth therapy has lagged, potentially due in part to issues associated with inconsistencies in production of therapeutic organisms. ^8^ Such inconsistencies are particularly evident in trials utilizing ova from the porcine whipworm, *Trichuris suis* (TSO).

Most clinical trials in the field of helminth therapy have been conducted with TSO, with mixed results. Some studies have shown success, ^9– 13^ while others failed ^14– 17^ or yielded equivocal results. ^18– 20^ Unfortunately, detailed methods describing production of TSO for clinical trials are absent from the literature, leaving questions regarding whether differences in production protocols may have affected results. ^8, 21^ As an example of the paucity of methodological details available, out of 12 trials with TSO that have been reported, ^9– 20^ none described the food supplied to the porcine hosts, and only four described any details regarding the porcine hosts. Three studies described “pathogen-free pigs”, ^10– 12^ without further detail, and another study described “barrier-bred Gottingen minipigs (miniature pigs)”. ^17^ Only two studies clearly describe viability of the ova used in the trials, ^9, 13^ and only one study described how the TSO were administered to the porcine hosts for production. ^10^

The information that has been published regarding production of TSO for clinical trials indicates that production methods have varied dramatically over time. For example, some trials employed ova produced *in vitro,* ^10– 12^ whereas others employed ova produced *in vivo.* ^9, 17^ At least two different washing methods for *in vitro* production have been described, one involving a potassium dichromate solution, ^12^ and the other involving a phosphate buffered solution. ^10, 11^ Storage of TSO for clinical trials has also been variable, with solutions described as saline, ^12^ phosphate buffered saline, ^10, 11^ sulfate-stabilized 0.05 mol/L H _2_SO _4_ (pH 1.0), ^17^ phosphoric acid buffer, pH 5, with 0.05% potassium sorbate preservative, ^9, 18^ with storage up to 2.5 years at 2-8 °C, ^9^ a solution at pH 5 with preservative, ^14^ or simply a “liquid”, ^15, 16^ depending on the study.

Clinical trials have been conducted with two nematodes, including TSO, ^9– 20^ a whipworm which imbeds itself into the gut epithelium, and larva of the hookworm *Necator americanus,* ^22– 28^ which penetrate the skin, burrow through the lungs, and then penetrate the gut epithelium of the small bowel. However, no trials have yet been reported with a cestode or with a species of helminth that remains within the lumen of the gut. Nevertheless, socio-medical studies evaluating the therapeutic potential of the cestode *Hymenolepis diminuta*, an organism that remains within the lumen of the gut, have shown that this organism may be effective, particularly for treatment of some allergic and neuropsychiatric conditions. ^21, 29^

We have previously described a detailed production method for *Hymenolepis diminuta* cysticercoids (HDCs), ^30^ the immature stage of *Hymenolepis diminuta* that can be cultivated in a laboratory and potentially used for helminth therapy. For such cultivation, adult *H. diminuta* are maintained in laboratory rats ( *Rattus norvegicus*), and the cysticercoids can be maintained in a variety of insects, although grain beetles ( *Tenebrio molitor*) are most often used in the laboratory. The method we previously described ^30^ was based upon a consensus among producers of the organisms for use in self-treatment-based helminth therapy. Under laboratory conditions, *H. diminuta* in a single laboratory rat produces sufficient eggs to load hundreds of beetles each week. The beetles, in turn, are readily produced by the thousands even in a small laboratory space. With each beetle yielding more than enough HDCs to treat the average patient for a week or longer, ^30^ HDCs could conceivably be produced in a cost-effective manner for the treatment of a large number of patients.

Although the HDCs live in a sterile environment in the extraintestinal space of their beetle hosts, bacterial contamination occurs when the HDCs are extracted from the peritoneal cavity of the host. Although bacteria present in grain beetles, an edible insect, are not hazardous to humans, growth of those microorganisms in storage solutions could potentially damage HDCs and lead to a rancid/unpalatable product. Further, bacterial growth would likely prevent the product from meeting FDA requirements for bacterial burdens in non-sterile pharmaceutical preparations. ^31^ Bacteria are readily removed from the HDCs by washing, but handling of individual HDCs during the washing process is time consuming to the point of prohibiting utilization of the organisms in sufficient quantities for clinical trials. ^30^

HDCs are morphologically complex, containing a juvenile helminth in their core, surrounded by a flexible, relatively fragile jelly-like layer and a tail-like structure that is not part of the juvenile helminth, but rather part of the jelly-like layer that is shed when the juvenile helminths excyst (emerge from the cysticercoid). ^32, 33^ The HDCs’ jelly-like outer coating, variable size, and flat, disk shaped geometry present unique challenges when attempting to separate the organisms from bacterial contaminants. Because of these physical properties, HDCs tend to adhere to a variety of surfaces and, the tail region in particular tends to break apart upon physical manipulation. Further, the tail region tends to have highly variable shapes, sometimes forming a long, narrow rod. These features present potential difficulties for the design and application of traditional mechanical filtration devices.

Here, we present a novel, scalable protocol for washing HDCs that preserves their morphology and viability. This includes the use of a patented device specifically designed to accommodate the organisms’ unique geometry and structural fragility.

## Methods

### Study design

The overall goal of the study was to develop a method of washing bacterial contaminants from HDCs on a large scale. A washing device utilizing liquid flow and gravity to create a steady state washing condition (see the section entitled “Washing device”, below) was envisioned to wash the HDCs en masse. Prior observations made in our laboratory ^30^ indicated that substantial differences in the size of HDCs exist, and it was hypothesized that these size differences may affect the organisms’ terminal velocities while falling through a solution, which would in turn affect the design parameters necessary for the washing device. With that in mind, the first phase of the study involved an assessment of (a) size variation in HDCs and (b) the corresponding speed (rate) at which the HDCs fall through solution. In this phase of the study, HDCs were considered the biological experiment unit. The only exception was when HDC size as a function of host was evaluated, in which case the host (grain beetle) was considered the biological experimental unit.

The second phase of the study involved the design, production and use of a device to wash multiple HDCs at a given time. The design and operation of the device was guided in large part by variations in HDC size and fall rate determined in the initial phase of the study. Since the intent of the work was not to evaluate biological variation in HDC size within the population, but rather to develop a method of isolating all HDCs within a population, hosts (grain beetles) or HDCs were selected non-randomly to ensure that a mixture of relatively small and large organisms were evaluated. In our previous work involving characterization of HDCs in considerable detail, ^30^ we observed that some secondary hosts (grain beetles) contain relatively large specimens, although this not typical. In our current studies, we selected HDCs that covered a wide range of sizes rather than evaluating “typical” HDCs in order to ensure that the method works for both typical and unusual HDCs. This approach facilitated determination of the relationships between HDC size and fall rate. However, to determine the association between individual hosts and HDC size, HDCs were necessarily selected for evaluation at random from a given host. Random selection was carried out by selecting the first organisms observed under the microscope. During selection, organisms which had a damaged (broken, not intact) tail-like structure were excluded and not evaluated.

For measurements of fall rates as a function of HDC size, HDCs were selected so that a broad distribution of sizes were included in the sample. Measurements were halted at n = 28 after it was determined that a sufficient sample set had been obtained to draw conclusions regarding the connection between sample size and fall rate. For assessment of HCD size within a given beetle, 10 to 13 HDCs were selected at random from each beetle for detailed measurement. Beetles were selected so that a broad distribution of HDC sizes was obtained, and experiments were halted when it was determined that a sufficient sample set had been obtained to draw conclusions regarding the association between beetle host and HDC size. For assessment of washing device function, beetles were selected at random and sufficient HDCs were extracted to load the device with between 80 and 120 HDCs for each run. The position of individual HDCs were assessed in the wash chamber in order to evaluate device function, and thus the number of HDCs Loaded in the device at a given time was limited by the ability to identify individual HDCs in the wash chamber: overcrowding in the chamber made it difficult to identify individual HDCs in the chamber.

Assessment of HDC viability after washing were conducted using 20 to 40 organisms, which was a convenient sample size for assessment in a single day. Since 100% of the HDCs were viable after washing, it was determined that experiments using HDCs without washing were unnecessary. No data points were excluded from the study.

The use of small sample sizes (10 to 13 organisms) for measurements of HDC size (2 dimensional surface area) as a function of the beetle host was selected as a starting point based on qualitative observations that HDC size tended to vary between hosts more so than within a given host. Since these observations were borne out while conducting the study (see Results), the use of larger samples sizes proved unnecessary. Correlations between continuous variables (size and fall speed) were evaluated using linear regression, and comparisons of size between different hosts were evaluated using ANOVA. GraphPad Prism 5 (by Dotmatics) was used for statistical analyses. A p-value < 0.05 was considered significant.

### Ethics approval

The use of laboratory rats as primary hosts for Hymenolepis diminuta was approved by the Duke University Animal Care and Use Committee (protocols A022-16-02 and A275-18-12). The Duke University Institutional Review Board (IRB) approved the application for patent coverage for the technology described in this manuscript (United States US Patent Application No. 17/296,908) by coauthor WP and, at the same time, the conduct of related research on human subjects by coauthor WP under the condition that co-author WP irrevocably refuse any potential financial benefits from the patent. That research on human subjects conducted by coauthor WP and colleagues (Duke IRB protocol Pro00045035) has been completed and published, ^8^ and is not the subject of the original work described in this manuscript.

### HDCs production and size measurement

HDCs were cultured as previously described using Sprague Dawley rats as primary hosts and *Tenebrio molitor* (grain beetles) as secondary hosts. ^30^ Under these conditions, laboratory rats host *H. diminuta* for the duration of their natural life span without apparent adverse effects from the tapeworms, and are sacrificed only when a humane endpoint is reached due to a general age-related decline in health or other age-associated issues (e.g. breast tumor development) unrelated to the experimental protocol. Clarified almond milk, produced as previously described, ^30^ was used for all procedures involving handling of isolated HDCs, unless otherwise indicated. Imaging software (Leica Application Suite V4.5; Leica Microsystems, Switzerland) and a Zeiss Stemi 2000-C microscope with a Leica MC 120 HD camera and a Zeiss KL 1500 LCD light source were used to photograph HDCs, and, ImageJ image analysis software (ImageJ 1.51k, Wayne Rasband, National Institutes of Health, USA) was used to quantify the size of individual HDCs. Heads and tails of HDCs were quantified separately (see results). Fall speed through solution was measured over a 20 cm distance in a 6 mm ID glass column using a stopwatch.

### Washing device

A glass washing device was fabricated (Carolina Research Glass Inc., Greensboro, NC) as shown in Figure 1 in order to wash HDCs of various sizes without contact with filters. The primary innovation is the “wash-hold” section (258 mm in length) with a diameter that increases from bottom (6 mm ID) to top (15 mm ID), as shown in the figure. As wash fluid is pumped from the inlet port up to the outlet port, appropriate adjustment of flow rates (see Results) results in the suspension of HDCs in the wash-hold section. HDCs can be eluted from the bottom of the device or, alternatively, a harvesting probe can be inserted into the wash-hold section ( Figure 1). For this study, all washed HDCs were harvested from the bottom of the device. The device has been patented (US patent 12,409,193 B2, September 9 ^th^, 2025). ^34^

**Figure 1.**
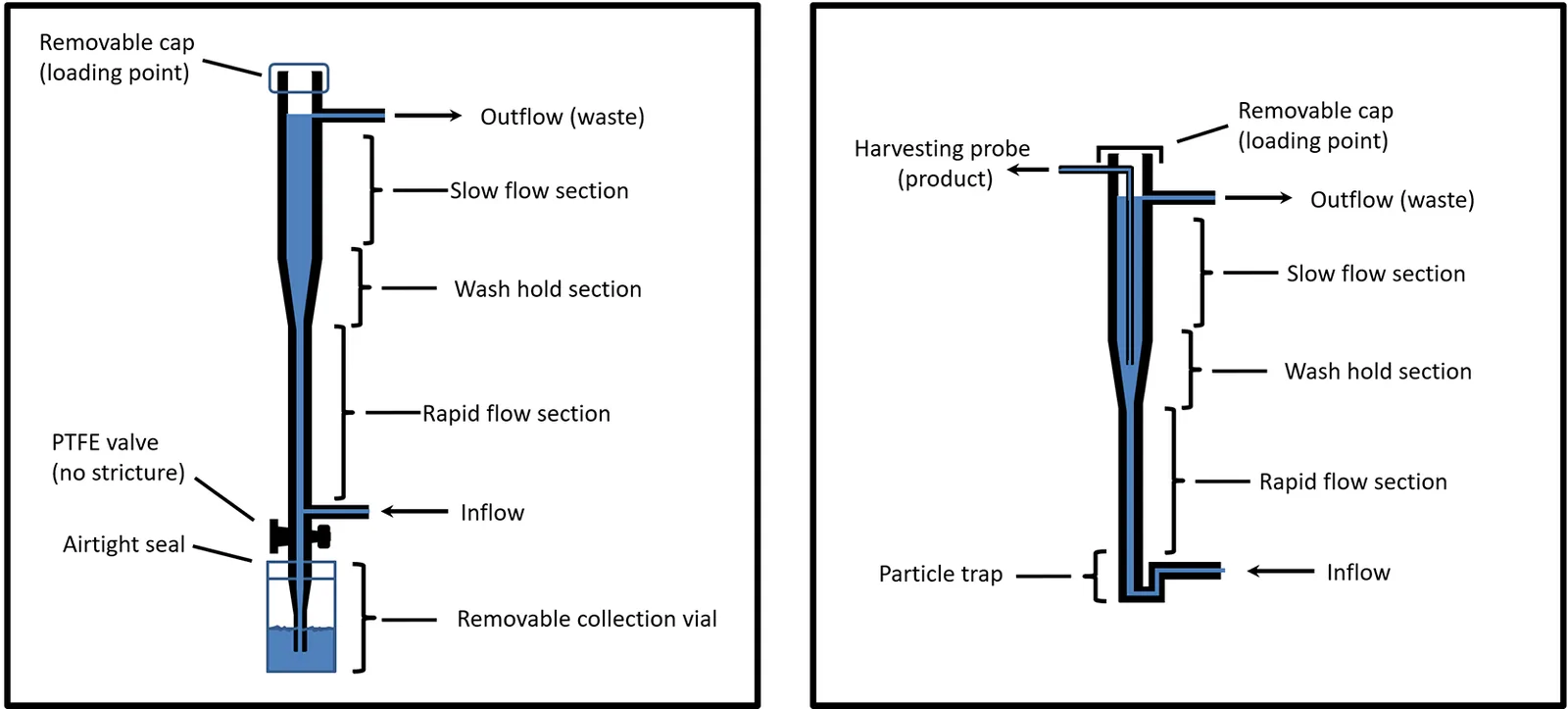
Schematic diagram of washing devices comprised of a single column washer that is designed to hold HDCs of different sizes in the “wash-hold” section, with a flow speed ranging from 0.04 cm/s - 0.2 cm/s, depending on the internal diameter of the washer. HDC’s can be harvested by decreasing the flow speed to > 0.04 cm/s in the rapid flow section, which enables elution of the HDCs from the bottom of the device on the left. On the right, insertion of a harvesting probe from the top into the wash-hold section is shown as an option. The harvesting probe allows for collection of any fraction of the HDCs in the wash-hold section rather than 100% of the HDCs in the wash-hold section, and allows the insertion of a particle trap in the event that any large contaminants are present in the HDC preparation.

### Viability assay

Viability of the HDCs was determined based on an excysting assay performed using an ibidi Stage Top Incubator (ibidi GmbH, Grafelfing, Germany) at 37 °C. For the assay, HDCs were placed in a 96 well plate (lumox® multiwell, 96, Sarstedtstr, D-51588 Nümbrecht Germany) using a Samco 231 disposable fine-tip pipette. Clarified almond milk was removed using the pipette, and 100 μl of 5 mg/ml pepsin, 60 mM HCl and 0.425% saline in 50% Tyrode’s saline buffer warmed to 37 °C was added to each well. The plate was then covered with an ibidi glass cover and observed at 37 °C for 10 minutes. The cover was then removed and the HDC’s were washed with approximately 300 μl of unbuffered 0.54% saline. The saline solution was removed, and 150 μl of 5 mg/ml trypsin with 5 mg/ml tauroglycocholate in 20 ml of Tyrode’s saline warmed to 37 °C was added to each well. Excysting was monitored for 90 minutes.

## Results

### Size of HDCs

The size of HDCs was found to vary over a considerable range based on approximations of the cross-sectional area of the organisms when lying flat on a dish in solution ( Figure 2). Head size was well correlated with tail size ( Figure 2), with the total estimated cross-sectional area (head size + tail size) varying over a 10-fold range ( Figure 3). The estimated area in the cross section was strongly dependent on the particular grain beetle ( *Tenebrio molitor*, the secondary host) from which the HDCs were extracted ( Figure 3) for reasons that are unknown.

**Figure 2.**
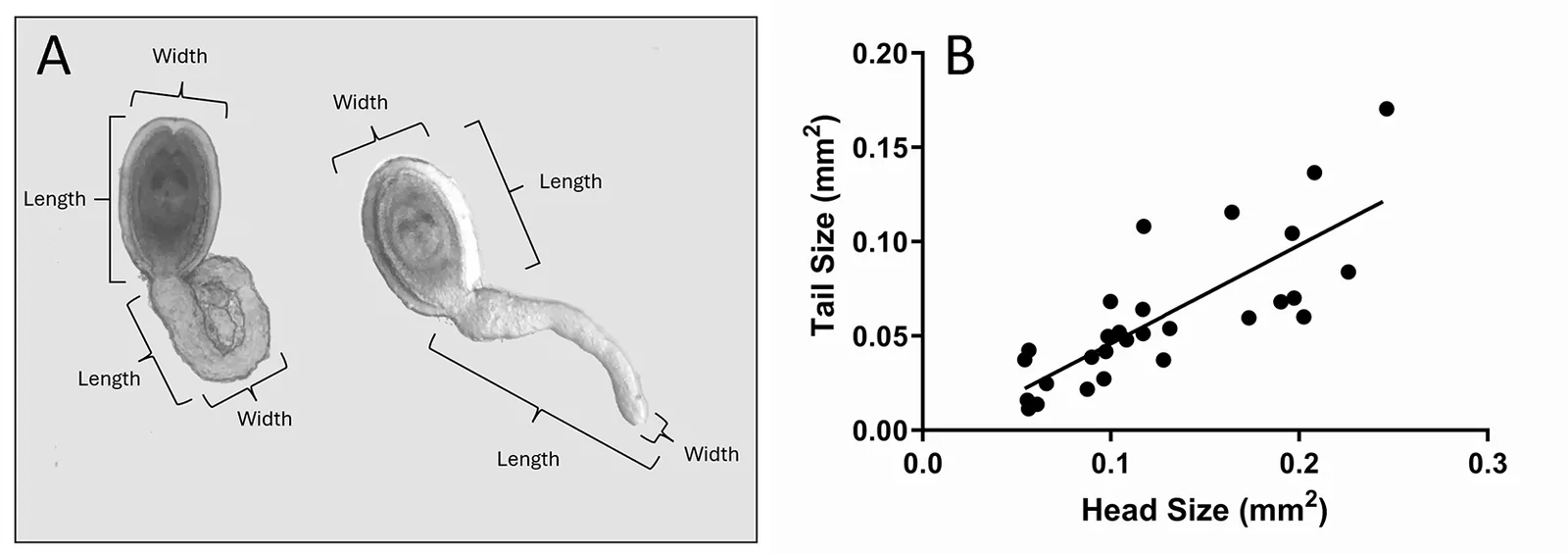
Measurements of HDCs to determine approximate head size and tail size. (A) The cross sectional area of each HDC was approximated by multiplying the length by the width for head and tails independently, then adding the areas together. Two HDCs, one with a compact (“ball shaped”) tail and one with a long tail, are shown for illustration. In the photo, background debris in the micrograph has been removed and the photos adjusted in size and contrast to match for the illustration. (B) Comparison between the two measurements, head size and tail size showed a positive correlation (C, n=28, r ^2^=0.6236, p<0.0001). The least-squares regression line is shown.

**Figure 3.**
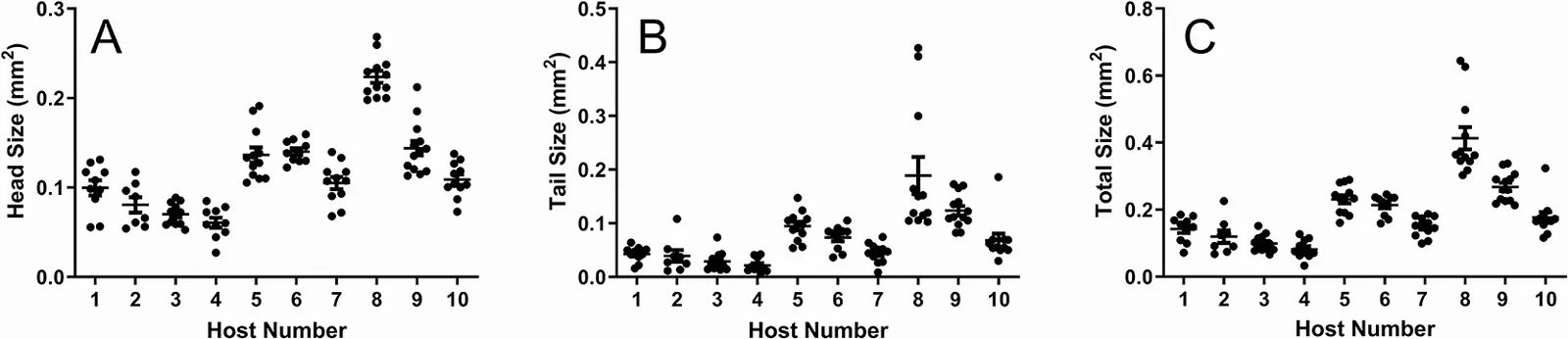
Variable size of HDCs as a function of the grain beetle ( *Tenebrio molitor*, the secondary host) from which the HDCs were extracted. Variable size of HDCs as a function of the grain beetle ( *Tenebrio molitor*, the secondary host) from which the HDCs were extracted. The head size (A), tail size (B), and total size (C) were assessed. n _1_=10, n _2_=8, n _3_=12, n _4_=10, n _5_=12, n _6_=10, n _7_=11, n _8_=12, n _9_=13, n _10_=11. With a one-way Anova test, p<0.0001 for A, B, and C. The bars indicate means and standard errors.

### Fall speed as a function of HDC size and shape

The fall speed of HDCs in solution varied over approximately a 5-fold range, and was dependent on helminth size as assessed by estimated cross-sectional area when lying flat on a dish in solution ( Figure 4). In addition to the effect of total cross-sectional area on fall speed, the effect of the shape of the tail on the fall speed was evaluated. The tail shape of HDCs is highly variable, with some having a long, extended tail, whereas others contain a more compact tail ( Figure 2, left panel). ^30^ Based on comparison of the fall speed of HDCs with straight tails to that of HDCs with more compact tails, the shape of the tail did not apparently affect the fall speed ( Figure 4D).

**Figure 4.**
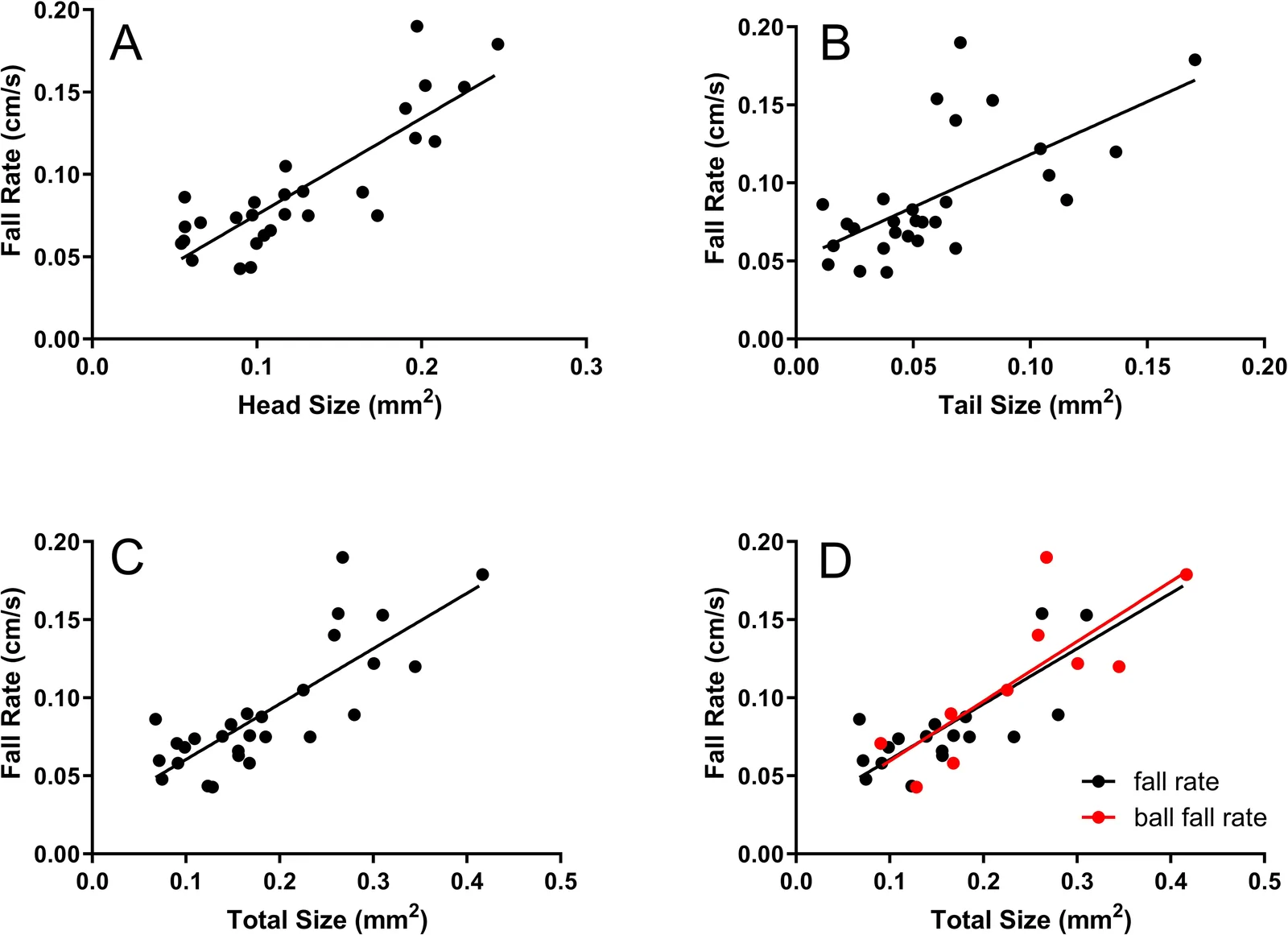
Variable fall rate as a function of head size (A, n=28, r ^2^=0.7120, p<0.0001), tail size (B, n=28, r ^2^=0.4139, p=0.0002), total size (C, n=28, r ^2^=0.6492, p<0.0001), and total size based on tail type (n _linear_=18, r ^2^=0.6492, p<0.0001, n _ball_=10, r ^2^=0.6326, p=0.0059). The least-squares regression lines are shown.

### Washing device function

The washing device described in the Methods ( Figure 1) effectively held HDCs in a steady position as the organisms “fell” through solution ( Figure 5). We observed that excessive crowding of HDCs in the device could result in some clumping and loss of organisms under the conditions used, so a loading capacity of about 200 HDCs was employed. The location of the organisms in the wash-hold section was linearly related to the flow rate ( Figure 5). After 90 minutes of washing, viability was assessed via excystation as described in the Methods. 100% of HDCs (31 out of 31 tested) successfully excysted immediately after washing. Furthermore, 100% (23 out of 23 tested) remained viable and excysted after being held in saline for an additional 24 hours post wash, demonstrating viability of the organisms following the washing procedure.

**Figure 5.**
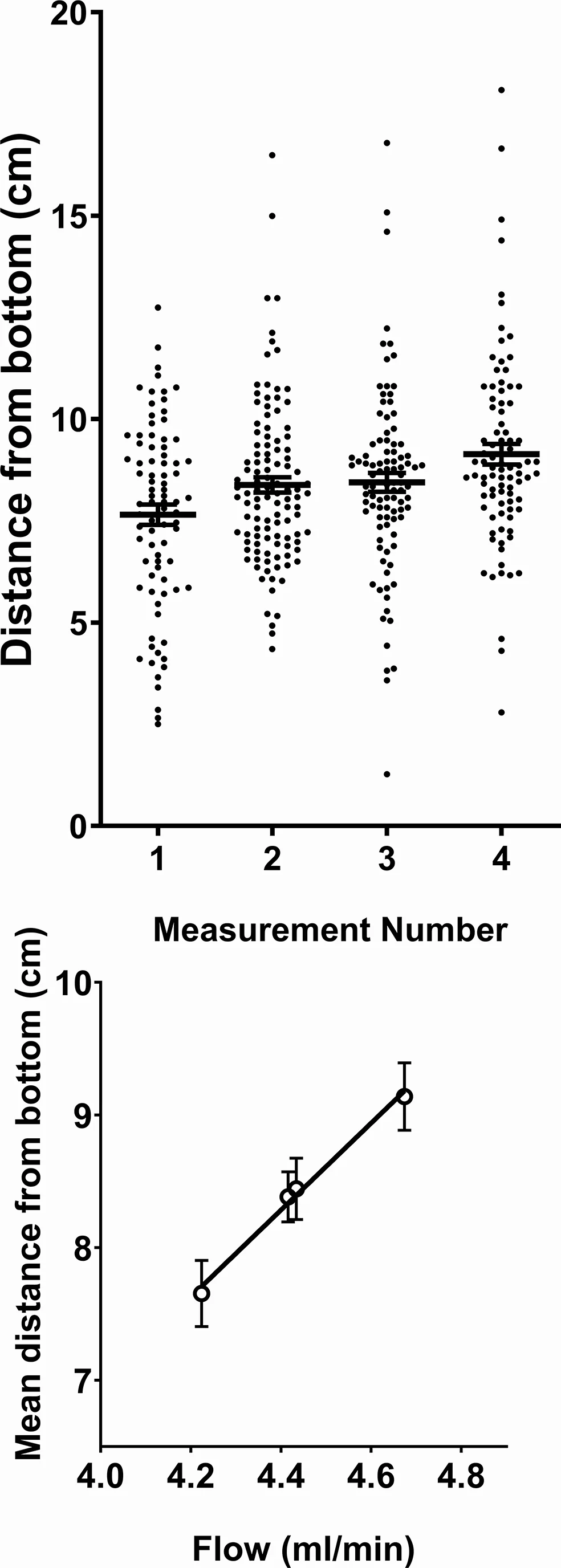
Position in the wash-hold section based on varying flow rates for each HDC. The distance was measured from the bottom of the wash-hold section. Four measurements were taken with three different flow rates (measurements 2 and 3 used the same flow rate). The number of organisms assessed in measurements one through four was 84, 111, 97, and 88, respectively. The top panel shows measures for individual HDCs, and the bottom panel shows average measures for each run as a function of flow rate. The mean distance from the bottom of the wash-hold section correlated strongly with the flow rate in a linear fashion (n=4, r ^2^=0.9918, p=0.0041). The least-squares regression line is shown, and the means and standard errors are indicated.

## Discussion

This paper presents a “no contact” washing procedure for HDCs, designed to effectively overcome limitations in the production of HDCs for therapeutic use that were previously described by our group. ^30^ This approach is sufficient to prepare large numbers of HDCs for clinical use in a manner that meets current FDA guidelines for bacterial contamination. Notably, HDCs remain viable after this washing procedure based on their ability to excyst. However, the viability of washed HDCs under long-term storage conditions remains an important area for future work.

As we discussed previously, ^30^ current FDA guidelines for oral biological therapeutics require that the number of CFUs be less than 100/ml in the final preparation, which is readily achievable with even a brief washing procedure carried out by a technician. However, washing in this manner was deemed impractical for large scale preparations due to excessive personnel time required for the process. ^30^ In contrast, given a very high estimate of an average dose of HDCs per person at 40/week, one device, run at one-hour intervals with 200 HDCs/run, would be sufficient to handle up to 200 patients. Further, the footprint of the device is less than approximately 10 cm ^2^, so multiple units of the device can conceivably be placed inside a single operating system and run simultaneously, in parallel, without creating an excessively large system.

The experiments described here were not conducted under sterile conditions such as those that might be required for preparation of HDCs for therapeutic use. Thus, although we have previously demonstrated ^30^ that even very brief washing is capable of removing the vast majority of bacterial contaminants from HDC preparations, and sometimes even all of them, it remains to be seen whether prolonged washing using the device described herein can remove all bacteria in most samples. Any remaining bacteria, if allowed to grow unchecked during storage, may render the HDCs rancid, and are likely to exceed the 100 CFU/ml regulatory allowance. Thus, if bacteria do adhere to some HDCs and are not removed by the washing system, the addition of chemical agents to the washing solution and/or the addition of antibiotics or antifungals for long-term storage may be considered.

An additional consideration is that an assessment of excysting may not accurately reflect the therapeutic potential of HDCs in humans; Some therapeutic potential might be lost during washing or storage regardless of whether the organisms can excyst. These issues are particularly relevant for industrial-scale production and application. In addition, if antibiotics or antifungals are required for storage, these may also affect the therapeutic potential of the HDCs. In the absence of validated biomarkers to assess the therapeutic function of HDCs *in vitro*, the only way to gain answers to the questions above will likely be through clinical trials. If indeed the cleaning and storage of HDCs proves to decrease the therapeutic efficacy of the organisms, alternative methods such as the automated extraction of HDCs directly from their secondary hosts may need to be developed in order to utilize fresh organisms for clinical use.

Despite all these limitations, Biome reconstitution remains a critical step towards alleviating underlying causes of chronic inflammatory diseases and normalizing immune function in Western society. ^2, 3^ However, as noted in the Introduction, utilizing a product in clinical trials for which the production method is highly variable and not publicly available imposes considerable limitations. First and foremost, without access to a production protocol, reproducibility by others is impossible. Second, if results are inconsistent, tracing the source of variability can be difficult. Troubleshooting has been particularly difficult in the field of helminth therapy, as companies have folded due to failure of clinical trials and academic investigators have retired without publishing detailed protocols. These challenges underscore the need for a fully disclosed method for the production of helminths used in clinical trials. However, this criterion presents challenges for commercial development, as patenting a species of helminth for clinical applications may prove difficult, ^35– 37^ leaving producers to rely on trade secrets for protection of their investments.

The quandary faced by investigators hoping to develop helminth therapy for widespread clinical use arises from a conflict between the considerable theoretical potential for helminths to normalize immune function and the practical challenge of protecting commercial interests necessary to procure funding of resource-intensive clinical trials from the commercial sector. One feasible solution is for governmental organizations and/or well-funded non-profits to conduct the clinical trials using a fully disclosed methodology. FDA approval of a product not protected by exclusive patent rights is conceivably an approach that could open the field for commercial development and yield dramatic benefits for both clinical practice and public health.

As pointed out in the section describing disclosures of competing interests, several of the coauthors have commercial interests in the field of helminth therapy, and thus have been faced with difficult decisions regarding the central issues discussed above: to fully disclose methods or to maintain trade secrets. Weighing heavily on this consideration is the fact that the first reports of the beneficial effects of helminths for autoimmunity ^38^^–^^41^ and for allergy ^42^ are now more than half a century old. Further, maintaining trade secrets, as has been done with trials involving TSO, has not led to widespread clinical use of TSO despite the fact that the initial studies using that helminth were published more than 20 years ago. ^10^^,^^12^ Further, reported differences between results in clinical trials and results from self-treatment suggest that problems associated with cleaning and storage of hookworms may be affecting trials involving those organisms. ^8^ With these issues in mind, it is the view of the authors both with and without commercial interests that, if helminth therapy is to become mainstream in the foreseeable future, it will be necessary to investigate the effects of the organisms in an objective manner that involves full disclosure of production methods, and with ample funding to evaluate the effects of different production methods.

## Preregistered data analysis

This study was not preregistered with a data analysis plan at an independent registry. The ARRIVE checklist is available at Parker, William (2026), “Helminth purification data v1”, Mendeley Data, V2, doi: 10.17632/dmht2t9tdr.2

## Data Availability

Data, graphs, and results of statistical tests are available at Parker, William (2026), “Helminth purification data v1”, Mendeley Data, V3, doi: 10.17632/dmht2t9tdr.3 ^43^
