# Supplementary material for: Isolation of Hymenolepis diminuta cysticercoids for therapeutic use: The need for fully disclosed methodology
Source: F1000Res. 2026 Sep 17;15:1334. Originally published 2026 Aug 8. [Version 2] doi: 10.12688/f1000research.186809.2 (PMC13675623; doi:10.12688/f1000research.186809.2)
Supplement: Peer review history

# Reviewer report: Reviewer response for version 2

**DOI:** 10.5256/f1000research.209925.r526356

**Published:** 2026-10-16

**Authors:** Prof. Dr. Friederike Ebner

**Affiliations:** 1 Department of Molecular Life Sciences, Chair of Infection Pathogenesis, School of Life Science, Technical University of Munich (TUM), Freising, Germany

The manuscript, “Isolation of *Hymenolepis diminuta* cysticercoids for therapeutic use: The need for fully disclosed methodology,” is a methods-focused research article that evaluates the applicability of a washing device for HDCs. The need for standardized procedures for obtaining parasitic material intended for therapeutic applications is not only clear but is convincingly demonstrated by the authors. The experiments conducted to validate the device are well designed and carefully performed.

 1. Although the present manuscript focuses exclusively on assessing material viability and morphological features and refers to a previous publication regarding bacterial decontamination, bacterial load should be assessed as a standard quality-control measure for each run or, at a minimum, in the key validation experiments. Furthermore, the authors might discuss whether the material properties of the device are compatible with the required sterilization procedures and applicable FDA regulatory requirements.

 2. If a single 90-minute washing step does not sufficiently reduce the bacterial load, would repeated or sequential washing procedures - for example, three washes over 24 hours - affect viability?

 3. Given that scalability appears to rely exclusively on operating multiple devices in parallel (horizontal scaling), could the authors clarify whether the fixed processing capacity of 200 HDCs per device can be increased through internal expansion or modification?

 4. The association between grain beetle characteristics and HDC size is an interesting finding, given the clarity and magnitude of the observed effect. Do the authors have data indicating whether host ( *Tenebrio molitor*) size or weight affects HDC size or morphology, and whether variability among HDCs can be attributed to differences in host size or weight?

 5. Minor suggestions regarding the figures:

 Figure 1 would benefit from the inclusion of the device dimensions.

 Figure 2 would benefit from the addition of a scale bar to the microscopy image.

 6. Very minor typographical issues:

 Please review and standardize capitalization in the Discussion and Methods sections.

 Please italicize *Hymenolepis diminuta* in the Ethics approval section.

Is the work clearly and accurately presented and does it cite the current literature?

Yes

If applicable, is the statistical analysis and its interpretation appropriate?

I cannot comment. A qualified statistician is required.

Are all the source data underlying the results available to ensure full reproducibility?

Yes

Is the study design appropriate and is the work technically sound?

Partly

Are the conclusions drawn adequately supported by the results?

Yes

Are sufficient details of methods and analysis provided to allow replication by others?

Yes

Reviewer Expertise:

Parasite Immunology, Infection and Mucosal Immunology, Biology of Parasites

I confirm that I have read this submission and believe that I have an appropriate level of expertise to confirm that it is of an acceptable scientific standard, however I have significant reservations, as outlined above.

# Reviewer report: Reviewer response for version 2

**DOI:** 10.5256/f1000research.209925.r522169

**Published:** 2026-10-09

**Authors:** Venkataramana Kandi

**Affiliations:** 1 Pratima Institute of Medical Sciences, Karimnagar, Telangana, India

The revised manuscript is scientifically sound and significantly improved, with clearer contextualization, expanded discussion of limitations, and stronger transparency. The contribution is valuable as a methodological framework, and it is acceptable for indexing.

Is the work clearly and accurately presented and does it cite the current literature?

Partly

If applicable, is the statistical analysis and its interpretation appropriate?

Yes

Are all the source data underlying the results available to ensure full reproducibility?

Partly

Is the study design appropriate and is the work technically sound?

Yes

Are the conclusions drawn adequately supported by the results?

Partly

Are sufficient details of methods and analysis provided to allow replication by others?

Partly

Reviewer Expertise:

NA

I confirm that I have read this submission and believe that I have an appropriate level of expertise to confirm that it is of an acceptable scientific standard.

# Reviewer report: Reviewer response for version 1

**DOI:** 10.5256/f1000research.206180.r510374

**Published:** 2026-08-22

**Authors:** Nikhilesh Joardar

**Affiliations:** 1 Dr Vishwanath Karad MIT World Peace University, Pune, Maharashtra, India

1. Where are the quantitative microbiological data demonstrating that washing actually removes bacterial contamination?
2. What is the HDC recovery/yield and does recovery remain unbiased across the substantial size distribution of HDCs?
3. Were beetles treated as the biological experimental unit, or were individual HDCs treated as independent observations despite multiple HDCs originating from the same host?
4. Why was an unwashed control unnecessary for the viability experiment, and how can 100% excystation after washing be interpreted without establishing baseline viability?
5. What evidence supports preservation of therapeutic/immunomodulatory potency rather than merely short-term excystation?

Is the work clearly and accurately presented and does it cite the current literature?

Yes

If applicable, is the statistical analysis and its interpretation appropriate?

I cannot comment. A qualified statistician is required.

Are all the source data underlying the results available to ensure full reproducibility?

Partly

Is the study design appropriate and is the work technically sound?

Partly

Are the conclusions drawn adequately supported by the results?

Partly

Are sufficient details of methods and analysis provided to allow replication by others?

Partly

Reviewer Expertise:

Host-pathogen interaction

I confirm that I have read this submission and believe that I have an appropriate level of expertise to confirm that it is of an acceptable scientific standard, however I have significant reservations, as outlined above.

# Response

**Published:** 2026-09-03

**Authors:** William Parker

**Affiliations:** Psychology and Neuroscience, The University of North Carolina at Chapel Hill, Chapel Hill, North Carolina, USA

We thank the editors of F1000Research for facilitating the rapid and extremely helpful review of our manuscript. We thank both Professor Kandi and Professor Joardar for their very kind evaluations of our manuscript and for their insightful comments. Both reviewers were very interested in what effects the washing process might have on the clinical efficacy of the helminths, and we agree this is a very important issue. We have addressed these and other comments from both professors as discussed in more detail below.

 The comments from the reviewers are noted in bold, with our responses in plain font.

**Reviewer 2:** **Professor Nikhilesh Joardar**, Dr Vishwanath Karad MIT World Peace University, Pune, Maharashtra, India 

  

1. **Where are the quantitative microbiological data demonstrating that washing actually removes bacterial contamination?**

This is an incredibly important topic that was not covered in the original manuscript. We thank Professor Joardar for asking about this issue, which will doubtless be of interest to the readers. In the revised manuscript, we point out that even a quick washing procedure is sufficient to remove the vast majority of bacteria so that the HDCs pass current FDA standards. This is based on previous work that is cited in the revised manuscript. However, since the studies described in this manuscript were not performed using sterile reagents and equipment, further studies will be required to know to what extent this device can be used to make preparations are or are not sterile. As we point out in the revised manuscript, sterility will be very important for long term storage of the organisms. If indeed some bacteria adhere to the HDCs, this is an issue that will have to be addressed.

1. **What is the HDC recovery/yield and does recovery remain unbiased across the substantial size distribution of HDCs?**

None of the HDCs are lost during the washing process. We did find that more crowding of HDCs in the device can result in clumping of the HDCs, so there are limits to the capacity of the device. This information has been added to the revised manuscript. At the same time, the yield from each beetle has, in our 15 years of experience, always been highly variable for reasons that are not understood. The beetles are “loaded” when they are deprived of moisture for about two days and then exposed to moistened fecal material containing eggs. This is apparently a highly variable process, and is difficult to control. However, grain beetles are easy to produce in mass quantities, so this is not an anticipated limitation of the use of HDCs for therapy.  

1. **Were beetles treated as the biological experimental unit, or were individual HDCs treated as independent observations despite multiple HDCs originating from the same host?**

As described in the revised document, the answer to this question depends on the experiment. If we were examining HDC size as a function of the host, then the beetle was treated as the biological experimental unit. However, for all other experiments, HDCs were treated as the experimental unit.

1. **Why was an unwashed control unnecessary for the viability experiment, and how can 100% excystation after washing be interpreted without establishing baseline viability? **

We make it clear in the revised manuscript that the purpose of testing viability after washing was to ensure that the washing process did not injure the organisms to the point that they could not excyst. By that measure, 100% of all organisms tested (absolute measure) after washing were still viable. Since viability cannot be greater than 100 percent in absolute terms, we confirmed that the washing process does not decrease viability. That being said, as mentioned by both reviewers, we do not know if the washing process affects the therapeutic benefits of the organisms.

1. **What evidence supports preservation of therapeutic/immunomodulatory potency rather than merely short-term excystation?**

This is a very interesting and incredibly important point, also raised by Professor Kandi. Please see point 4b raised by Professor Kandi for a detailed discussion of this issue.

# Reviewer report: Reviewer response for version 1

**DOI:** 10.5256/f1000research.206180.r510366

**Published:** 2026-08-20

**Authors:** Venkataramana Kandi

**Affiliations:** 1 Pratima Institute of Medical Sciences, Karimnagar, Telangana, India

The manuscript fills a critical gap in helminth therapy research by introducing an open‑source, reproducible protocol for isolating *Hymenolepis diminuta* cysticercoids (HDCs). Its rationale is firmly grounded in the broader context of biota restoration and the pressing need for standardized production methods that can be adopted across laboratories. The work is particularly timely, as it directly engages with ongoing debates surrounding reproducibility and transparency in helminth therapy trials, offering a methodological framework that strengthens both scientific rigor and translational potential.

 1. The manuscript introduces a patented washing device as an innovative contribution, yet it requires stronger contextualization by comparing this approach with established purification techniques such as mechanical filtration and chemical washing. Furthermore, the claim of offering an “open‑source methodology” appears contradictory in light of the patenting of the device. The authors should clarify how the method can remain freely accessible and adoptable by other researchers despite intellectual property restrictions, ensuring transparency and reproducibility are not compromised.

 2. The study employs relatively small sample sizes, with 28 observations for fall rate and 10–13 beetles per group for size measurements. While the justification provided is reasonable, the discussion should more fully address whether these numbers are adequate to support broader generalizability of the findings. In addition, although the excystation assay is an appropriate measure of viability, the authors should acknowledge its limitations as a surrogate endpoint, since it does not directly capture therapeutic efficacy in clinical or translational contexts.

 3. The manuscript appropriately acknowledges Institutional Review Board (IRB) and Institutional Animal Care and Use Committee (IACUC) approvals, which is commendable. However, the dual role of the authors as both inventors and chief executive officers of companies with direct financial interests introduces potential conflicts of interest. This issue warrants more explicit discussion within the limitations section to ensure transparency and to contextualize how such affiliations may influence interpretation of the findings.

 4. The discussion appropriately underscores the importance of reproducibility in helminth therapy research, but it would benefit from further elaboration on how the proposed method could be scaled for industrial-level production and aligned with regulatory approval pathways. In addition, the authors should address whether washed *Hymenolepis diminuta* cysticercoids (HDCs) retain their immunomodulatory properties during long‑term storage, as this represents a critical translational question with direct implications for therapeutic viability and clinical application.

Is the work clearly and accurately presented and does it cite the current literature?

Partly

If applicable, is the statistical analysis and its interpretation appropriate?

Yes

Are all the source data underlying the results available to ensure full reproducibility?

Partly

Is the study design appropriate and is the work technically sound?

Yes

Are the conclusions drawn adequately supported by the results?

Partly

Are sufficient details of methods and analysis provided to allow replication by others?

Partly

Reviewer Expertise:

Medical Microbiology, Clinical Microbiology, Infectious Diseases

I confirm that I have read this submission and believe that I have an appropriate level of expertise to confirm that it is of an acceptable scientific standard, however I have significant reservations, as outlined above.

# Response

**Published:** 2026-09-03

**Authors:** William Parker

**Affiliations:** Psychology and Neuroscience, The University of North Carolina at Chapel Hill, Chapel Hill, North Carolina, USA

We thank the editors of F1000Research for facilitating the rapid and extremely helpful review of our manuscript. We thank both Professor Kandi and Professor Joardar for their very kind evaluations of our manuscript and for their insightful comments. Both reviewers were very interested in what effects the washing process might have on the clinical efficacy of the helminths, and we agree this is a very important issue. We have addressed these and other comments from both professors as discussed in more detail below.

 The comments from the reviewers are noted in bold, with our responses in plain font.

**Reviewer 1. Professor Venkataramana Kandi**, Pratima Institute of Medical Sciences, Karimnagar, Telangana, India

**The manuscript fills a critical gap in helminth therapy research by introducing an open‑source, reproducible protocol for isolating *Hymenolepis diminuta* cysticercoids (HDCs). Its rationale is firmly grounded in the broader context of biota restoration and the pressing need for standardized production methods that can be adopted across laboratories. The work is particularly timely, as it directly engages with ongoing debates surrounding reproducibility and transparency in helminth therapy trials, offering a methodological framework that strengthens both scientific rigor and translational potential.**

 We are grateful to Professor Kandi for his kind description of our manuscript and his understanding of the work we are conducting.

**1a. The manuscript introduces a patented washing device as an innovative contribution, yet it requires stronger contextualization by comparing this approach with established purification techniques such as mechanical filtration and chemical washing.**

 We thank Professor Kandi for these ideas. In the revised manuscript, we point out that, in our experience, the HDCs tend to adhere to a variety of surfaces, break apart upon manipulation, and have odd shapes, particularly in the tail region. These properties may preclude mechanical filtration, although we have not conducted extensive tests to probe the utility of mechanical filtration. In addition, we add Professor Kandi’s idea that chemicals could be added to the wash solution to aid in cleaning, although presumably this would only be done if the washing system as currently described proves to be ineffective at complete sterilization.

**1b. Furthermore, the claim of offering an “open‑source methodology” appears contradictory in light of the patenting of the device. The authors should clarify how the method can remain freely accessible and adoptable by other researchers despite intellectual property restrictions, ensuring transparency and reproducibility are not compromised.**

 Professor Kandi’s point is very well taken. The patent only covers the USA, so the device is now “open source” everywhere except in the USA. Coauthor WP, in consultation with officials at Duke University, had previously concluded that a patent may be helpful in driving commercial interest in the USA. However, the device will, at the very least, require a license to produce in the USA, so it is not technically open source in that country.  With that in mind, the term “open-source” has been replaced by “fully disclosed” throughout the manuscript. This is a much better description of the views presented in the manuscript, and we thank Professor Kandi for pointing this out.

**2. The study employs relatively small sample sizes, with 28 observations for fall rate and 10–13 beetles per group for size measurements. While the justification provided is reasonable, the discussion should more fully address whether these numbers are adequate to support broader generalizability of the findings.**

 In our previous work involving characterization of HDCs in considerable detail, we observed that some secondary hosts (grain beetles) contain relatively large specimens, although this is not typical. In our current studies, we selected HDCs that covered a wide range of sizes rather than evaluating “typical” HDCs. We did this to ensure that the method works for both typical and unusual HDCs. This information is now described in the revised manuscript.

**2b. In addition, although the excystation assay is an appropriate measure of viability, the authors should acknowledge its limitations as a surrogate endpoint, since it does not directly capture therapeutic efficacy in clinical or translational contexts.**

 This issue as well as other issues that might affect therapeutic efficacy are now discussed in more detail in the revised manuscript. See larger discussion below under point 4b.

**3. The manuscript appropriately acknowledges Institutional Review Board (IRB) and Institutional Animal Care and Use Committee (IACUC) approvals, which is commendable. However, the dual role of the authors as both inventors and chief executive officers of companies with direct financial interests introduces potential conflicts of interest. This issue warrants more explicit discussion within the limitations section to ensure transparency and to contextualize how such affiliations may influence interpretation of the findings.**

 It is a very interesting proposal to describe the effects of conflicts of interest in the text of the document, outside of the section of the manuscript normally assigned for this purpose. We have added a final paragraph of the manuscript describing our thoughts on this issue. While some of us have academic backgrounds without commercial interests, others do indeed come at this from a commercial point of view. Yet, we are all in agreement that a lack of disclosed methodology and lack of funding to test different methodology has led to decades without significant progress. In a nutshell, as a collective of both scientists and commercial producers of helminths, we have come to the conclusion that the only way forward in the foreseeable future is to utilize the scientific method, which means that methods must be fully disclosed. We thank the reviewer for this very interesting suggestion, and hope the readers of the manuscript will find this new part of the manuscript interesting.  

**4a. The discussion appropriately underscores the importance of reproducibility in helminth therapy research, but it would benefit from further elaboration on how the proposed method could be scaled for industrial-level production and aligned with regulatory approval pathways.**

 We thank Professor Kandi for suggesting that we include this very practical information. In the revised manuscript (second paragraph of discussion), we discuss the current FDA guidelines, the relative number of patients that might be covered by a single device, and the potential for multiple copies of the device to be included in a single operating system and run in parallel.

**4b. In addition, the authors should address whether washed *Hymenolepis diminuta* cysticercoids (HDCs) retain their immunomodulatory properties during long‑term storage, as this represents a critical translational question with direct implications for therapeutic viability and clinical application.**

 We agree with Professor Kandi, and have included this issue in the revised manuscript. First, it is unknown whether washed HDCs will have the same therapeutic potential as unwashed (fresh) HDCs. Indeed, as pointed out in the revised manuscript, washing of hookworms prior to storage may have had a detrimental effect on trials involving that species.  Second, it is unknown to what extent total (complete) removal of bacteria can be obtained using the washing device described. We appreciate these concerns.  In the original version of the manuscript, we had noted that clinical trails may be needed to evaluate the effectiveness of washed HDCs. However, we did not discuss the potential need for additional anti-microbial measures, and we did not discuss the potential effects of these measures on therapeutic activity. These issues have been addressed in the revised manuscript. In addition, we propose a course of action in the event that washing and storage does prove detrimental to the clinical efficacy of HDCs.

                 The bottom line is that clinical trials are going to be needed to sort these issues out, which will require FDA-approved protocols and substantial resources. We appreciate the concerns of the reviewers, and acknowledge that finding answers to the questions raised will require extensive effort.
